# Diagnostic Accuracy of Ultrasound in Detecting Retained Products of Conception: A Study from a Tertiary Care Hospital in Karachi, Pakistan

**DOI:** 10.7759/cureus.3564

**Published:** 2018-11-08

**Authors:** Hina Iqbal, Muhammad Salman Khan, Aeman Muneeb, Waseem A Mirza

**Affiliations:** 1 Radiology, Aga Khan University, Karachi, PAK

**Keywords:** retained products of conception, ultrasound pelvis, histopathology

## Abstract

Introduction

Retained products of conception (RPOC) are a known complication after abortion or childbirth. To improve clinical evaluation and avoid unnecessary surgery, transvaginal scan is performed in suspected cases. However, both RPOC and blood clots appear isoechoic on ultrasound, and false positives can lead to unnecessary intervention. In this study, the ultrasound findings have been correlated with the histopathology (as a gold standard) to determine the diagnostic value of sonography in the detection of RPOC in postpartum or post-abortion patients.

Methods

This cross-sectional study was carried out to determine the diagnostic accuracy of ultrasound in the detection of the retained products of conception in relation with the histopathological findings at the Department of Radiology, Aga Khan University Hospital (AKUH), Karachi.

A total of 193 patients with suspicion of RPOC undergoing a transvaginal scan in the Department of Radiology, AKUH, were enrolled. The study was conducted for a period of 12 months from October 2014 to October 2015.

Results

Our results yielded that out of 193 cases, 113 cases (87.05%) had histopathology positive for RPOC, while 107 (55.44%) RPOC cases were identified by ultrasound as having RPOC. The mean endometrial thickness of the patients included in the study was 13.5 mm. According to our results, ultrasound has a sensitivity of 75.22%, specificity of 72.50%, a positive predictive value (PPV) of 79.44%, a negative predictive value (NPV) of 67.44%, and a diagnostic accuracy of 74.09%.

Conclusion

Transvaginal ultrasound is a modality that can be used for early diagnosis of the retained products of conception including fetal parts and could prove to be lifesaving. However, the operator and equipment variables need to be looked at, and a uniform criterion needs to be agreed on.

## Introduction

Retained products of conception (RPOC) are the second most common cause of postpartum bleeding, after uterine atony [1]. RPOC are a treatable complication after delivery or termination of pregnancy [1]. Common clinical signs/symptoms that lead to suspicion of RPOC are secondary postpartum hemorrhage (PPH), either alone or associated with pain, fever or both [2].

The reported incidence of RPOC is around 1% of all deliveries, and it is considered to be the most common reason for hospital readmission in the postpartum period [3]. The diagnosis of RPOC presents a major clinical challenge, which relies on different clinical symptoms and signs as well as sonographic assessment [3]. Ultrasonography has been used extensively, in addition to clinical evaluation, for the diagnosis of RPOC [3].

However, ultrasound-detected intrauterine findings following termination of pregnancy or delivery are quite variable, and residual trophoblastic tissue or blood clots can appear alike [3]. A false diagnosis of RPOC inevitably leads to curettage with the possibility of complications [3]. A previous study concluded that ultrasound is a useful diagnostic tool as opposed to clinical criteria alone, despite the diagnostic challenges when trying to differentiate RPOC from similar appearing pathology [4]. Another study concluded that a combination of clinical and ultrasound are the best in terms of accuracy [5].

In Pakistan, unintended pregnancies are on the rise, and according to one study [6], the abortion rates almost doubled between 2002 and 2012. A large majority of these are illegal abortions, done by untrained providers in unsafe facilities [6] and likely to lead to complications such as RPOC.

This study was carried out to determine the diagnostic accuracy of ultrasound in patients with suspected RPOC and thus establish if ultrasound can be useful for diagnosis in women with clinical suspicion of RPOC.

## Materials and methods

This prospective cross-sectional study was carried out to determine the diagnostic accuracy of ultrasound in the detection of RPOC in relation with the histopathological findings at the Department of Radiology, Aga Khan University Hospital (AKUH), Karachi.

A total of 193 patients with suspicion of RPOC undergoing a transvaginal scan in the Department of Radiology, AKUH, were enrolled. The study was conducted from October 2014 to October 2015 for a duration of 12 months.

Inclusion criteria

The inclusion criteria were as follows: female patients of childbearing age referred to our department for a transvaginal scan (either from the clinic or from the emergency department) with clinical suspicion of RPOC and patients who underwent dilation and curettage at AKUH and the final histopathology diagnosis was available.

Exclusion criteria

The exclusion criteria comprised patients who were managed conservatively and those who underwent dilation and curettage outside AKUH.

Sampling size and technique

A non-probability consecutive sampling technique was used. With a prevalence of 5% [7] and a confidence interval of 95%, the calculated sample size was at least 193.

Data collection

Patients referred for sonography at the radiology department, who met the inclusion criteria, were enrolled in the study after taking informed consent. Patients' relevant history and examination were recorded on standardized data collection forms. Transvaginal sonography was performed on a Toshiba Xario/Aplio system with either convex transducer (7.2 to 14 MHz) or endocavity transducer (5.0 to 7.5 MHz). Images were acquired by senior residents/faculty and recorded on Picture Archiving and Communication system (PACS). Surgical evacuation of the uterus was performed by OBGYN (obstetrician-gynecologist) and the specimen was sent for histopathology. The histopathology results were obtained from the electronic medical records by the principal investigator. Data were entered and analyzed using the Statistical Package for Social Sciences (SPSS) program version 20.0.

## Results

**Figure 1:**
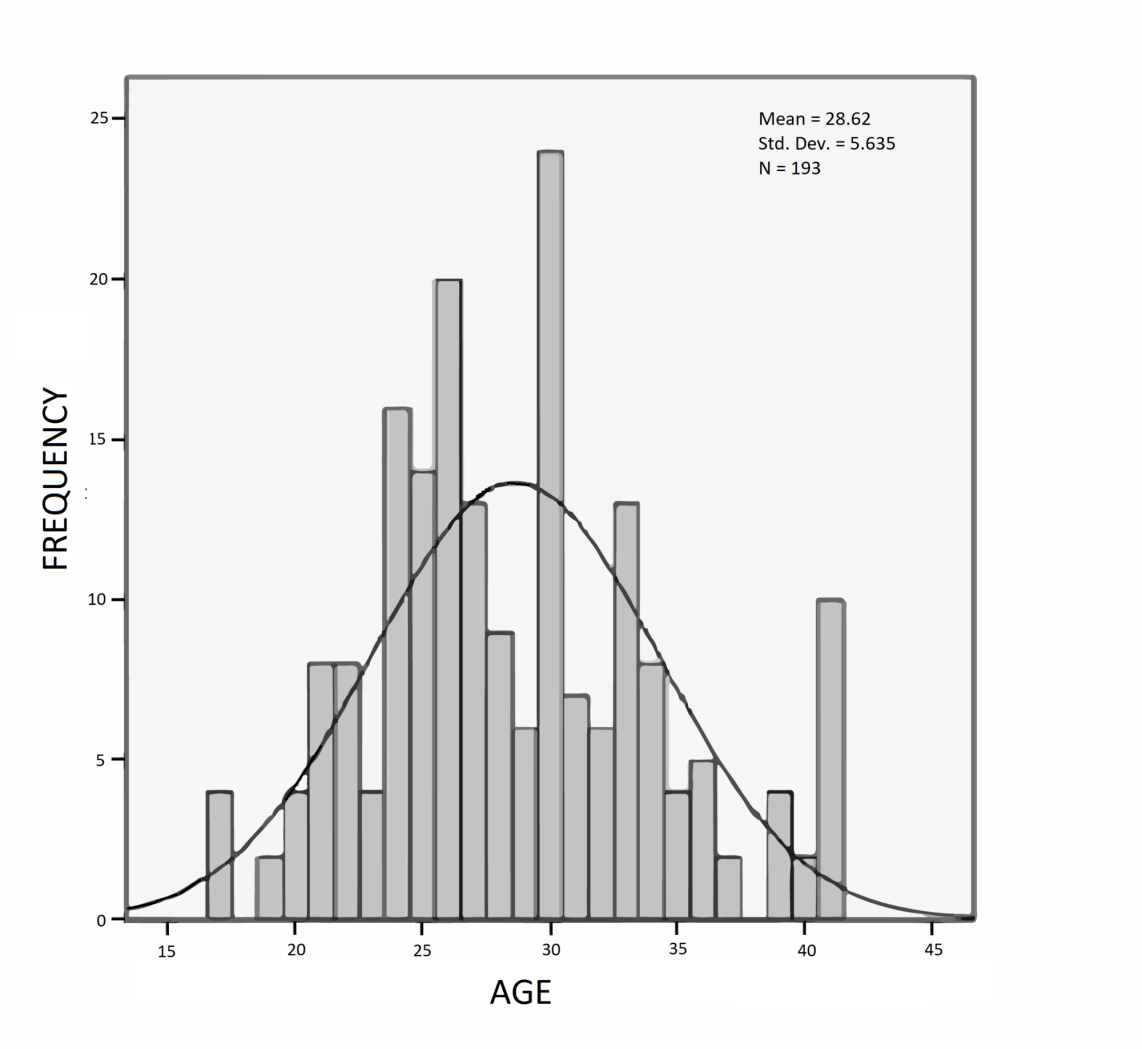
Histogram showing frequency distribution of age of the study participants

The mean age of the patients included in the study was 28.62 years (SD = 5.64 years). The age range was from 17 to 42 years (Figure 1).

**Figure 2:**
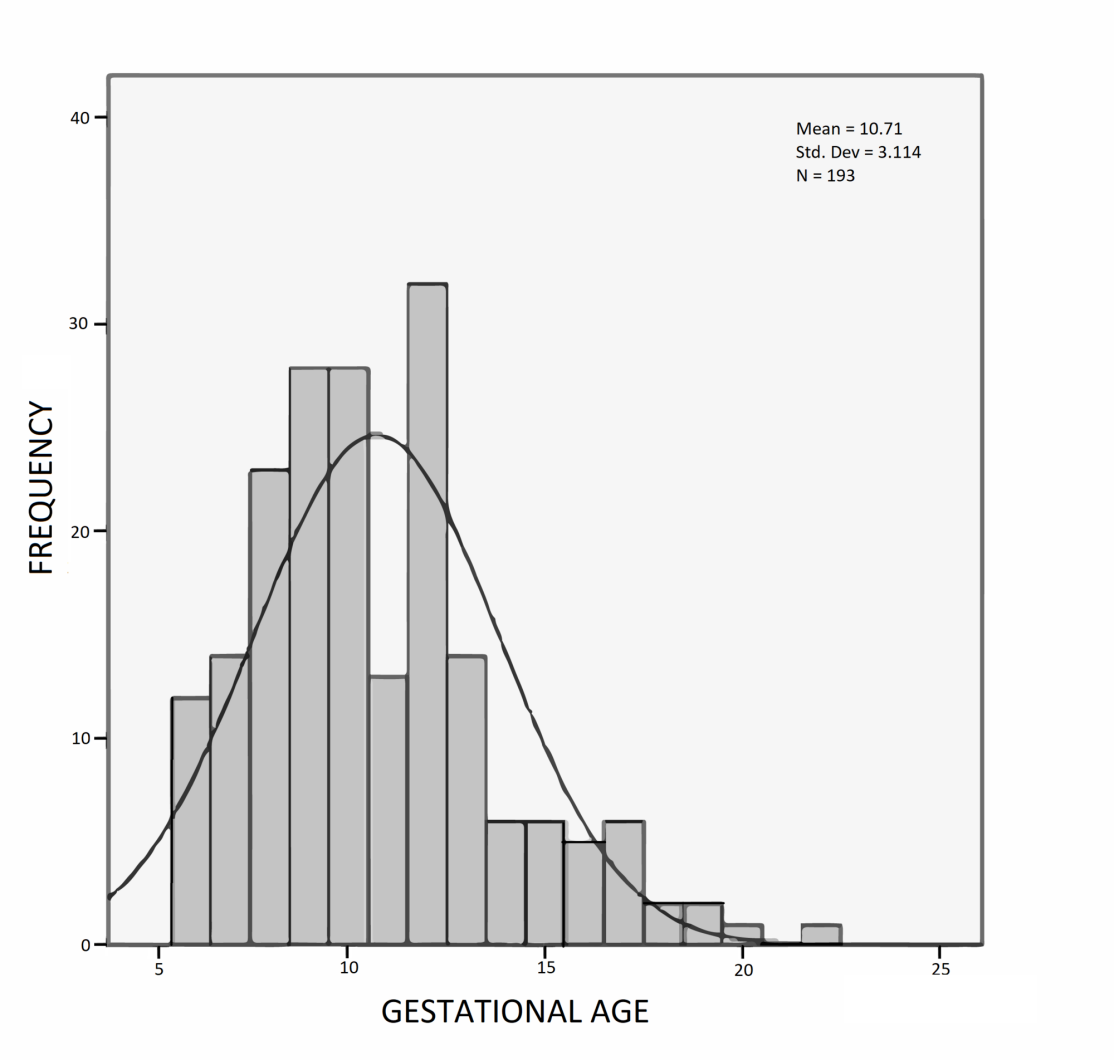
Histogram showing frequency distribution of gestational age

The mean gestational age of the patients included in the study was 10.7 weeks (SD = 3.11 weeks). The gestational age range was from 7 to 22 weeks (Figure 2).

**Figure 3:**
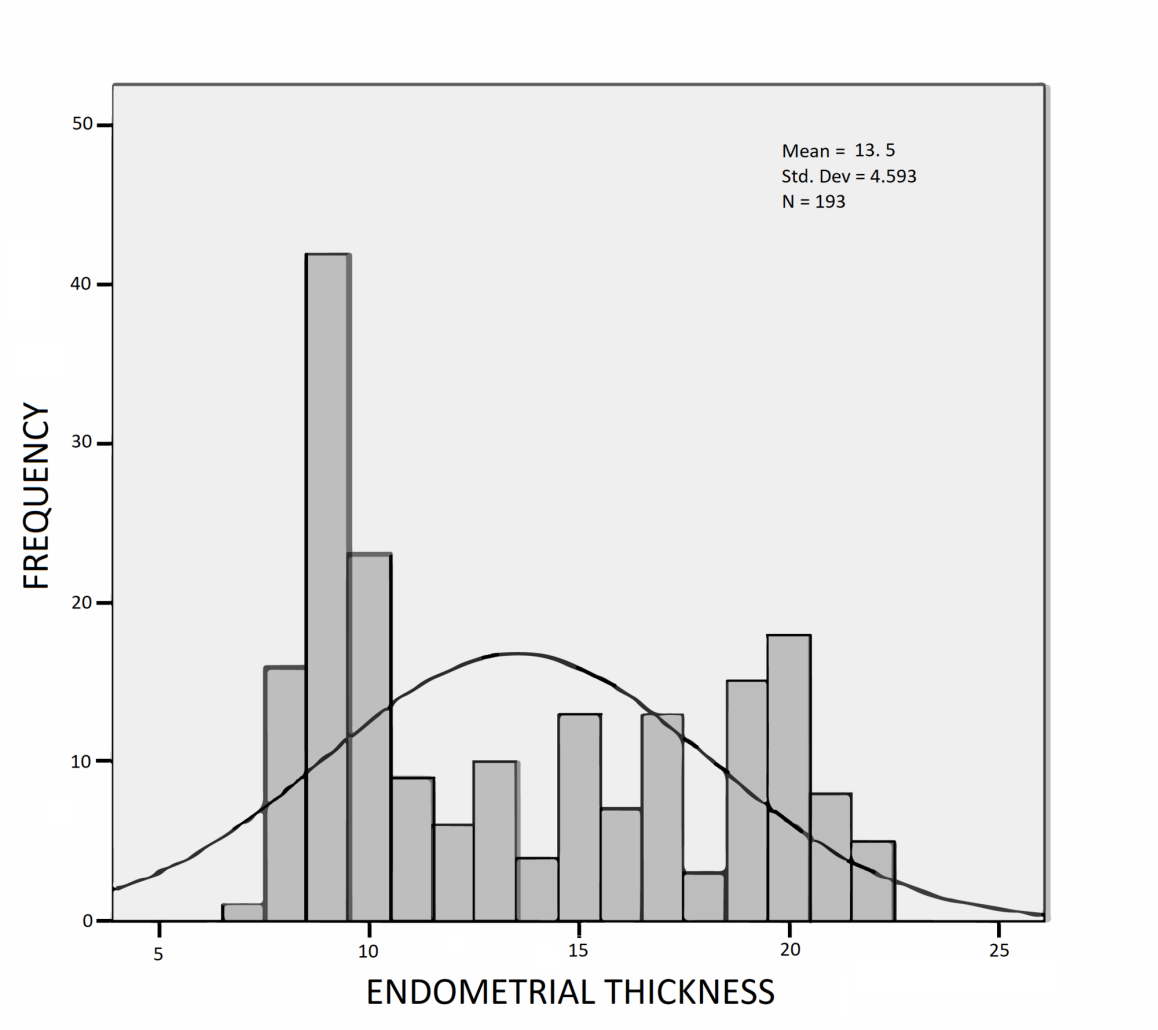
Histogram showing frequency distribution of endometrial thickness

The mean endometrial thickness of the patients included in the study was 13.5 mm (SD = 4.5 mm). The endometrial thickness range was from 6 to 22 mm (Figure 3).

Out of the 193 cases, 113 cases (58.5%) were identified by histopathology as having RPOC, while 107 (55.44%) were identified by ultrasound.

According to the results of this study, ultrasound has a sensitivity of 75.22%, specificity of 72.50%, a positive predictive value (PPV) of 79.44%, a negative predictive value (NPV) of 67.44%, and a diagnostic accuracy of 74.09% (Table 1).

**Table 1:** Ultrasound and histopathology correlation 2 x 2 showing the correlation between ultrasound and histopathology findings RPOC: retained products of conception

		Histopathology for RPOC		Total
		Positive	Negative	
Ultrasound	Positive	85 (79.4%)	22 (20.6%)	107 (100%)
	Negative	28 (32.6%)	58 (67.4%)	86 (100%)
Total		113 (58.6%)	80 (41.5%)	193 (100%)

## Discussion

The ultrasound finding of hyperechoic or mixed echogenicity mass within the endometrial cavity is the most sensitive predictor of diagnosing RPOC [8]. If no mass is seen and the endometrial thickness is less than 10 mm, RPOC are extremely unlikely [8]. Sawyer et al. found that the measures of endometrial thickness and volume were not reliable for diagnosing RPOC [9]. Similarly, Durfee et al. found that complex fluid or endometrial thickness greater than 10 mm were not considered reliable measures either [8]. Color Doppler flow is considered relatively helpful with an increased vascularity correlating with RPOC [10]. While blood clots may be the most commonly confused diagnosis with the retained products, other conditions such as arterio-venous malformations (AVMs) of the uterus or invasive moles may also appear similar on ultrasound [1]. Since AVMs and moles have high vascularity and RPOCs can also demonstrate vascularity, they are differentials for each other. However, an absence of flow is a negative predictor for AVMs and mole but not RPOCs. In our study, the findings of a discrete mass in the uterine cavity were considered positive ultrasound evidence of retained products.

A 2010 study in south Iran [5] calculated 87% sensitivity, 41% specificity, 71% PPV, 65% NPV, and a diagnostic accuracy of 70% ultrasound alone.

While our sensitivity (75%) and NPV (67%) were comparable, our specificity (72%) and PPV (79%) were considerably higher than Karimpour [5], showing that our study has a lower false positive rate (high specificity) and therefore, subjects who tested positive on ultrasound were more likely to have RPOC (high PPV).

Possible explanations for these differences could include a different patient population, operator skill/experience and a difference in equipment. Another reason could be the difference in the ultrasound criteria used to define RPOC. While our definition was based on the finding of a discrete mass, Karimpour [5] looked at a variety of ultrasound findings including echogenic material in the uterus, endometrial lining (thickness and regularity), etc. Our diagnostic accuracy (of 74%) was also higher than the previously calculated 69.7% [5].

Another similar study by Matijevic et al. [4] reported a 98% sensitivity and 33% specificity for the detection of RPOC using the predefined sonographic criteria (including the presence of a mass >10 mm with echogenic patterns in the endometrium and low resistive index on Doppler), while Chopra et al. [11] reported sensitivity (92%), specificity (60%), PPV (87.3%), and NPV (71.4%) using the criteria of endometrial echogenicity alone (thickness was measured if echogenicity was normal). Esmaeillou et al. [12] examined both color Doppler and gray-scale ultrasonography and used a variety of criteria to define RPOC; they found that while a criterion of endometrial thickness greater than 1 0mm gave a perfect (100%) sensitivity and NPV, the addition of color Doppler criteria improved the diagnostic accuracy overall. Using the presence of a hyperechoic mass as the sole criteria, they found a 94% sensitivity and a 77% specificity, both of which are higher than the findings of our study, probably due to factors similar to the ones discussed above.

As the proportion of illegal abortions is increasing day by day, especially in the lower socioeconomic population, accurate detection of RPOC on ultrasound can lead to reduced morbidity and mortality due to the timely intervention in confirmed cases. Additionally, avoiding unnecessary intervention when RPOC is ruled out also decreases complications and costs associated with curettage. Our results, similar to previous studies, show that transvaginal scan is a reliable method for confirming the presence of the retained products within the uterus as confirmed on the subsequent histopathological analysis; however, equipment and operator factors need to be studied. A standard uniform criterion also needs to be established for the imaging diagnosis of RPOC.

## Conclusions

Ultrasound is a modality that can be used for the early diagnosis of RPOC. As the incidence of illegal abortions increases, leading to a higher incidence of complications, an imaging modality such as ultrasound, which is available even in the remote areas of the country, could prove to be lifesaving. Additional benefits are that sonography is a noninvasive technique without any potentially harmful side effects and can be performed on portable ultrasound devices. However, since ultrasound is dependent on operator and equipment dependent, establishing the uniform criteria would improve utility.
